# A Novel Alternative Methods for Decalcification of Water Resources Using Green Agro-Ashes

**DOI:** 10.3390/molecules26226777

**Published:** 2021-11-10

**Authors:** Safaa El-Nahas, Abdulrahem S. Arafat, Hanan Salah El Din, Abdulrahman G. Alhamzani, Mortaga M. Abou-Krisha, Hesham M. Alsoghier

**Affiliations:** 1 Chemistry Department, Faculty of Science, South Valley University, Qena 83523, Egypt; Safaa33@yahoo.com (S.E.-N.); hanan.eldin@yahoo.com (H.S.E.D.); mmaboukrisha@imamu.edu.sa (M.M.A.-K.); hgmohmed@gmail.com (H.M.A.); 2 Red Sea Company for Drinking and Waste Water, Hurghada 84511, Egypt; abdoarafat46@yahoo.com; 3 Chemistry Department, College of Science, Imam Mohammad Ibn Saud Islamic University (IMSIU), Riyadh 11623, Saudi Arabia

**Keywords:** hardness removal, agro-ash, agro-waste, removing calcium ions, SWOT analysis

## Abstract

The strategic idea in this work was to increase pH values by employing natural alkali sources (i.e., HCO_3_^−^ and CO_3_^2−^) from four tested agro-ashes as an alternative to chemicals (i.e., lime or soda ash). The considerable proportion of carbonates and bicarbonates in the investigated ash products had remarkable features, making them viable resources. All ash materials showed a significant ability for Ca ion elimination at high initial Ca ion concentrations. A slight quantity of ash (10 g/L) was sufficient for usage on very hard water contents up to 3000 ppm. Finally, the tested agro-ash was free of cost. Furthermore, unlike other conventional precipitants, such as NaOH, Ca(OH)_2_, NaHCO_3_, Na_2_CO_3_, and CaO, they are cost effective and ecologically sustainable. There is no need to employ any additional chemicals or modify the agro-ash materials throughout the treatment process. The benefits of the manufactured ash were assessed using a SWOT analysis.

## 1. Introduction

No living being can survive without water. Therefore, water is fundamental for human life. Water is essential in cultivation, cleaning, mining, and industrial usage in addition to drinking purposes [1]. Water scarcity, climate change, and pollution are the most essential environmental topics to be concerned about in the 21st century [2]. As water resources are limited, one of the biggest problems facing the world these days is the water crisis. Water scarcity arises when the demand for freshwater exceeds accessible water. Thus, great efforts are needed for better management of available water resources to reduce water shortages, and easy access to a fair amount of clean water is one of the main goals of the Sustainable Development Goals (i.e., Goal 6). Wastewater from the industrial sector is discharged by direct or indirect methods and easily pollute water supplies in the surrounding environment. Globally, approximately 300–400 million tons of pollutants (toxic sludge, heavy metals, organic waste, and solvents) are deposited every year into the surrounding environment via industrial plants [1]. Management of industrial water is a vital topic in sustainable development. Taking into account that, in accordance with a country’s industrial progress, approximately 20% of the freshwater widely consumed is used for industrial purposes. In developing countries, this percentage falls to less than 10%, while in developed countries it doubles. Internationally, this ratio is expected to increase as a result of the growing population and industrial development [3]. Improvement in water-use efficiency can minimize the expected gap between water demand and supply to approximately 40% in order to find a solution to water scarcity by 2030. Notably, the growth in water demand is projected particularly for the production of energy and electricity via hydroelectricity. In addition, water employed in industrial processes is mainly used in cooling systems [4]. Challenges facing the water sector were highlighted as an important issue related to economic activities and water services. One of the widespread and costly problems in many industrial processes is scaling formation. The elimination of calcium ions from aqueous solutions is considered a rich area for study in industrial uses [5]. Hard water means there are high soluble amounts of Ca and Mg ions in the water supply. The presence of calcium ions in water or wastewater is the reason for numerous operational problems with extensive economic implications [6]. Continuous usage of hard water forms scale on the interior walls of domestic and industrial machinery. The augmentation of the scale formation leads to the blockage of pipes. Moreover, scales decrease the flux rate in pipes, decline heat transfer performance, in some cases cause malfunction of the equipment, and in the worst case leads to closure of the industrial unit [7,8]. Scales are precipitates of calcium ions as carbonate, phosphate, or sulphate, but they largely form as calcium carbonate [8,9]. Currently, the employment of evaporation technology has achieved great interest in the treatment of wastewater, as it can be utilized for extremely intense wastewater pollutants. Many evaporation systems, such as multistage flash (MSF), multiple effect evaporation (MEE), thermal vapor compression (TVC), and mechanical vapor recompression (MVR), are largely employed [10]. Among these techniques, MVR is more desirable because of its economical, energy-saving, easy operation, and low-temperature evaporation process. MVR has been broadly employed in various manufacturing industries such as the chemical of purification food, petrochemicals, wastewater purification, and desalination [10,11]. The water or wastewater used by the MVR technique should contain low concentrations of calcium and magnesium ions, below 100 ppm and 500 ppm, respectively. A decline in scale ions has become essential as an earlier step in such evaporation techniques as a result of low thermal conductivity and a reduction in heat transfer in the heater or boiler of scales [11]. Therefore, the membrane fouling efficiency used for the discharged wastewater of flue gas desulfurization (FGD) employed in thermal power plants (such as wet scrubber technologies) is negatively affected by high levels of hardness ions such as Ca and Mg. Hence, eliminating Ca and Mg ions from the FGD wastewater treatment are essential steps for facilitating the ordinary operation of the membrane system [12]. Most of the industrial sector attempts to precipitate Ca ions from solutions such as CaCO_3_. This can be accomplished by increasing the pH with the addition of a base (e.g., NaOH or Ca(OH)_2_) as a conventional remedy for hardness problems in the occurrence of sand granules [9]. Several approaches have been published for the removal of ions causing scales (i.e., Ca and Mg ions) using different methods such as CaO/Na_2_CO_3_, precipitation by oxalic acid and phenoxy acetic acid [11], selective nanocomposite electrosorption electrode [13], ion exchange resins (the alkyl phenoxy acetic acid derivatives) [11], injection of CO_2_ gas [14], microbial carbonate precipitation process (MCP) [15], phosphosilicate glass [5], wheat straw ash (WSA) and rice husk ash (RHA) [16], brine pre-treatment waste of soda ash plant such as an alkali source and CO_2_ [17], and synthetic zeolite [18]. In the scope of the present circumstances, it is important to achieve sustainable development for future generations, utilizing the principles and concepts of green chemistry [1]. It is time to change our approaches by using eco-friendly applications to reduce the quantity of waste (Goal 12 of the SDGs). Agricultural wastes are undesirable substances and are freely available. By 2030, the recycling perspective of waste materials assessed in tons should be increased. The environmentally sound management of chemical and hazardous waste for healthy lifestyles via the use of clean water, air, safe food, and a sustainable ecosystem helps to reduce consumption of primary resources [19]. In 2013, burned biomass ash waste reached 480 million tons and will increase for years throughout the consumption of biomass as fuel [20]. The current environmental criteria emphasize the need to promote recycling processes to convert unattractive waste into usable materials [21]. This work took advantage of the naturally present inorganic minerals in biomass for reuse as alkali sources to increase the pH value of aquatic solutions. The carbonate and bicarbonate content of ash materials have many advantages and benefits. They are readily available, inexpensive as waste, and have basic nature in water. They are suitable precipitants for removing calcium ions from any aqueous solutions without any addition of a base (i.e., NaOH, Ca(OH)_2_, or KOH). Thus, the ash material can be easily used as an alternative to lime/soda ash or Ca(OH)_2_. Ash materials are not recognized as toxic substances, and they are a product from edible plants. Thus, the goal of this study was the green removal of calcium ions from water supplies using different agro-ash materials as an economic source of natural minerals.

## 2. Experimental

### 2.1. Collection of Raw Materials for Ash

**Figure 1:**
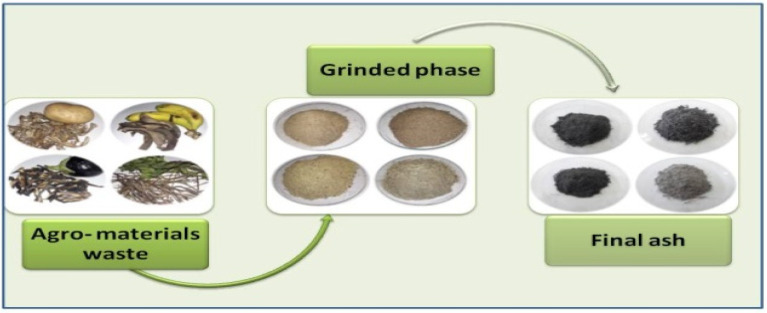
Method for preparation of agro-ash.

Potato peel (*Solanum tuberosum*), banana peel (*Musa*), eggplant peel (*Solanum melongena*), and the stem of mint (*Mentha*) were the domestic wastes. First, they were washed thoroughly with deionized water to remove dust, color, and other impurities. Then, they were cut separately into small pieces. After washing, they were dried in an electric oven at 80 °C for 4 h until they reached a stable mass. The dried samples were crushed to make a homogenous phase. The dried wastes were burned in a muffle furnace at 500 °C for 6 h. The resulting ashes were milled, sieved, and stored in desiccators prior to use in bench-scale experiments or for analysis of their physical characterization. Figure 1 represents the method used for the preparation, and the obtained ashes yields were approximately 10.5%, 25%, 11.3%, and 13.7% of the waste dry matter for potato peel, banana peel, eggplant peel, and mint stem, respectively. The amount of ash yield was comparable to data published by others [16]. 

### 2.2. Characteristics of Fly Ash Samples

The prepared ash samples (i.e., Po-ash is Potato peel Ash, BA-ash is Banana peel Ash, Eg-Ash is Eggplant ash, and Min-Ash is Mint stem ash) were characterized by various spectroscopic techniques, such as FTIR (Fourier transform infrared), and measurements were conducted using a (Nicolet) Magna-FTIR–560 (USA) with the KBr technique and powder X-ray diffraction analysis (XRD) of the ashes using a Brucker Axs-D8 Advance Diffractometer (Belgium) at ambient temperatures in the 2Θ range between 10 and 70°. The morphology and elemental analyses of the ash samples were characterized using an EDX (Model FEI INSPECT S50) operating at 20 kV. The desired pH values of the solutions were measured using a precision pH meter (Model PHS-3C).

### 2.3. Chemicals and Materials

The stock solution of Ca (II) was 4000 mg/L. HCl (0.5 M) or NaOH (0.5 M) solutions were used. Chemical precipitants, such as NaOH, Ca(OH)_2_, NaHCO_3_, Na_2_CO_3_, and CaO, were purchased from (Sigma–Aldrich (USA), Fluka (UK), and Merck (Germany)) and used directly.

### 2.4. Hardness Removal Experiment

The examined parameters for the removal efficiency of calcium ions by tested ash-materials were the individual effects of the initial concentrations (1000–4000 ppm), pH (2–10), contact time (1–180 min), and adsorbent dosage (1–14 g/L), while the operating temperature was fixed at 25 °C. For each analysis, 0.5 g of ash/50 mL of Ca (II) solution (1000 ppm) was put into a 250 mL flask and mixed. The shaker speed was set to 200 rpm for stirring for 1 h at room temperature. After shaking, the residue ash was separated from the solution by filtration, and the final Ca^2+^ concentration was determined according to the standard method in the 23rd Edition, no: 3500-Ca B [22]. Moreover, pH, TDS, and conductivity were measured for *C_o_* and *C_e_* of Ca ions. The percentage of Ca ion removal was calculated according to Equation (1).

(1) $$\%\;Removal=\frac{\left(C_{o}-C_{e}\right)}{C_{o}}\times 100$$

where *C_o_* and *C_e_* are the initial and the final Ca ion concentrations at equilibrium (mg·L^−1^), respectively.

### 2.5. Studying Other Chemical Precipitations Compared with Agro-Ash

Several laboratory examinations were carried out to compare various conventional methods. In this section, five common chemical precipitants were utilized, such as NaOH, Ca(OH)_2_, NaHCO_3_, Na_2_CO_3_, and CaO as well as the two mixtures of CaO and Na_2_CO_3_ and Ca(OH)_2_ and Na_2_CO_3_ for the elimination of Ca ions from solutions compared with tested ago-ashes.

## 3. Results and Discussion

### 3.1. XRD Analysis of Ash Materials

**Figure 2:**
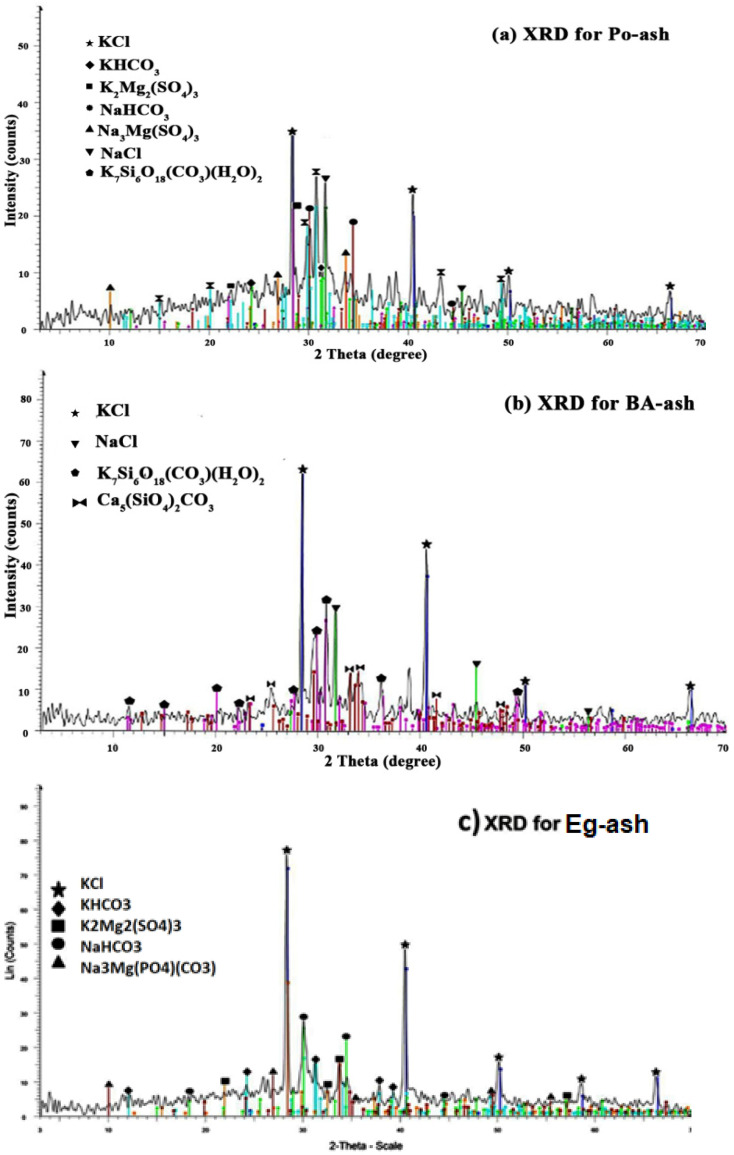

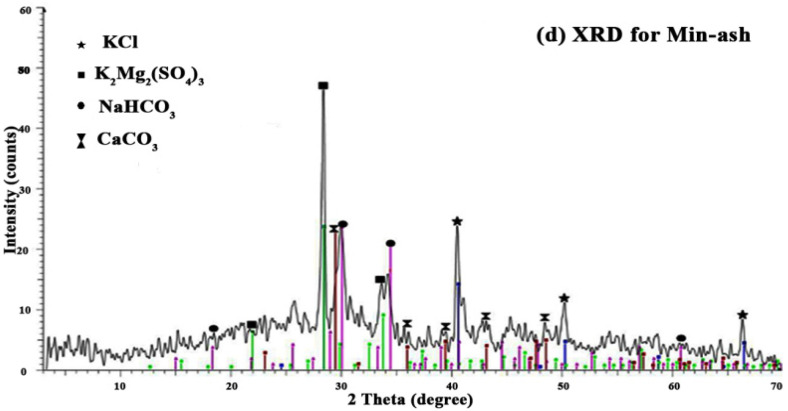
The XRD patterns for the characteristics of the agro-ash compositions: (**a**) Po-ash, potato peel ash; (**b**) BA-ash, banana peel ash; (**c**) Eg-ash, eggplant peel ash; (**d**) Min-ash, mint stem ash materials.

The XRD results confirmed the exact mineral composition of the prepared agro-ashes. The data from the XRD patterns for the tested agro-ashes are illustrated in Figure 2 and Table 1. The major constituent in all of the ash samples was sylvite (KCl). Individually, Po-ash constitutions comprised seven minerals: nahcolite (NaHCO_3_), kalicinite (KHCO_3_), langbeinite (K_2_Mg_2_(SO_4_)_3_), bradleyite (Na_3_Mg(PO_4_)CO_3_), scawtite (Ca_7_(Si_6_O_18_)(CO_3_)H_2_O), sylvite (KCl), and halite (NaCl). While the composition of the Eg-ash observed had only five constituents: kalicinite (KHCO_3_), langbeinite (K_2_Mg_2_(SO_4_)_3_), bradleyite (Na_3_Mg(PO_4_)CO_3_), nahcolite (NaHCO_3_), and sylvite (KCl). Min-ash had four constitutions: nahcolite (NaHCO_3_), langbeinite (K_2_Mg_2_(SO_4_)_3_), calcite (CaCO_3_), and sylvite (KCl). BA-ash showed four compositions: scawtite (Ca_7_(Si_6_O_18_)(CO_3_)H_2_O), spurrite (Ca_5_(SiO_4_)_2_CO_3_), sylvite (KCl), and halite (NaCl). Calcium silicates were observed in other types of agriculture ash materials derived from wood [23,24]. The revealed data show most ash materials had major contents of KCl compared to NaCl as observed by another researcher [25]. Furthermore, many agricultural ash materials mainly contain multiple silicate compounds and chlorides, oxides, carbonates, sulphates, and phosphates for common metal ions such as Ca, K, Mg, and Na [26,27]. We can conclude from the XRD results that all tested ashes varied in the amount of carbonate and bicarbonate; in addition to NaHCO_3_ and KHCO_3_, Po-ash, BA-ash, and Eg-ash contained other types of carbonates such as Na_3_Mg(PO_4_)CO_3_, Ca_7_(Si_6_O_18_)(CO_3_)(H_2_O)_2_, and Ca_5_(SiO_4_)_2_CO_3_.

### 3.2. SEM-EDX Analysis of Ash Materials

**Figure 3:**
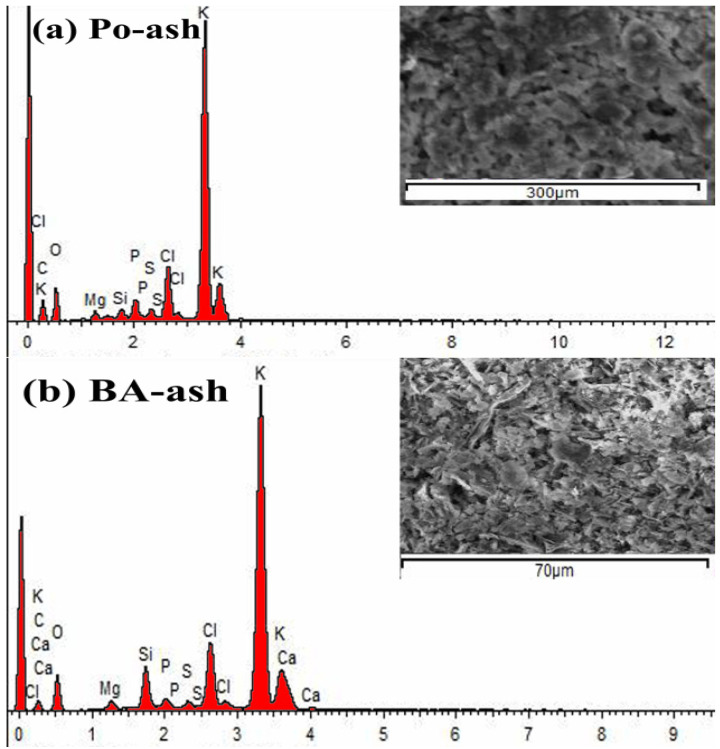

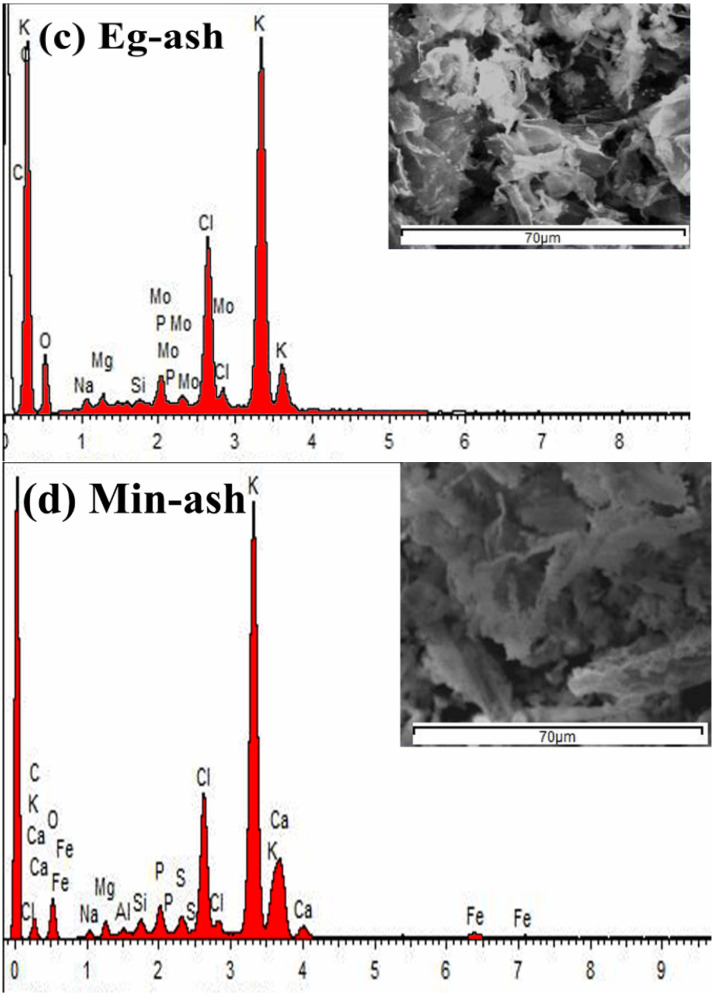
EDX–SEM analyses for (**a**) Po-ash; (**b**) BA-ash; (**c**) Eg-ash; (**d**) Min-ash materials.

EDX analysis provided the elemental composition and constituent of the ashes as demonstrated in Figure 3. Noticeable variations in the major elements for the tested ash samples are illustrated in Table 2. High potassium contents were observed in all studied ash samples following the order BA-ash > Min-ash > Po-ash > Eg-ash. While Na showed minor levels compared to K contents as observed in [25]. Po-ash and Eg-ash had small concentrations of calcium compared to BA-ash and Min-ash. However, the tested ashes showed that the carbon content varied from 11–25% in the following order: Eg-ash > Po-ash > Min-ash > BA-ash. In fact, C and N are usually oxidized during ignition and distorted to a gaseous component, but the amount of C was still present in ash in indefinite amounts due to the fact of incomplete burning [28]. The presence of other elements, such as Mg, P, S, Cl, and Si, was tolerable, as the ashes resulted from plant sources. In addition, the oxygen content varied from 36.9% to 69.6%. The EDX analysis was compatible with XRD data for the existing elements in the ash compositions and their mineral phases. Ash reactivity and its elemental distribution were strongly impacted by the biomass structure [29]. Understanding the exact chemical composition of produced ash materials will be helpful in developing the reactivity of agriculture biomass as low-priced materials for water treatment.

SEM images provide additional details regarding surface information of the tested materials at extremely high magnifications. The structures of the tested ash materials, as shown in the SEM photos, displayed a heterogeneous texture, and the particles had an irregular shape with crevices-like structure similar to others [30,31,32,33].

### 3.3. FTIR Analysis

**Figure 4:**
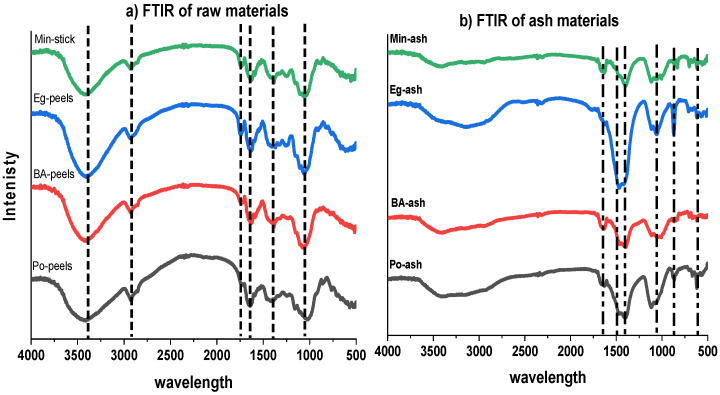
FTIR spectra: (**a**) raw materials; (**b**) ash materials.

FTIR (Fourier transform infrared spectroscopy) is a sensitive technique for recognizing active functional groups of compounds to identify the composition of organic or inorganic materials [34]. Table 3 signifies the distinct functional forms in the tested raw and ash materials. Agricultural waste materials usually include some essential components such as cellulose, hemicelluloses, lignin, lipids, proteins, simple sugars, water hydrocarbon, and starch [35]. The obtained spectra in Figure 4a exhibited various absorption bands for tested raw agriculture waste, indicating the complicated nature of the examined agriculture wastes. The FTIR spectra showed a broad band at approximately 3401 cm^−1^, representing bonded –OH groups [33,36]. While the band observed at approximately 2915 cm^−1^ could be assigned to the stretching C–H bond of the CH_2_ group [33,37,38,39]. Furthermore, the bands at approximately 1749 cm^−1^ were assigned to the carboxyl groups (–COOH) of the peels’ structure [33,37,38]. Moreover, the band at approximately 1064 corresponded to the stretching vibration of Si–O and Si–O–Si [33,36]. Furthermore, the band at 1639 cm^−1^ was assigned to the water bending vibration of the H–O–H bond [39,40]. It seems that these types of functional groups are similar to other waste materials reported in [37,38,41,42]. The spectra of agro-ash materials are illustrated in Figure 4b, and the bands of agricultural waste at approximately 3401 and 2915 cm^−1^ completely disappeared in the agro-ash spectra. However, new peaks appeared at approximately 1647, 1450, 1104, 1061, 863, and 617 cm^−1^. The peak at approximately 1400–1450 cm^−1^ was characteristic of the C=O stretching bond of CO_3_^2−^ [27,43,44,45]. Whereas the intensive band at 1104 cm^−1^ corresponded to PO_4_^3−^ [43,46]. The peak at approximately 1061 cm^−1^ was attributed to the presence of SiO_4_^2−^ [27,33,36,46,47]. However, the recorded band at approximately 863 cm^−1^ was distinctive to metal bonded to oxygen [42]. Mainly, the peak at approximately 617 cm^−1^ belonged to the bending S–O of sulphate (SO_4_^2−^) [43,48]. All the indicated peaks for carbonate, phosphate, sulphate, silicate, and metal oxide were present in the form of kalicinite, langbeinite, bradleyite, nahcolite, calcite, scawtite, and spurrite of the tested ash materials as previously confirmed by XRD analysis.

### 3.4. Assessment of the Ability of the Tested Ash Materials to Remove Ca Ions

**Figure 5:**
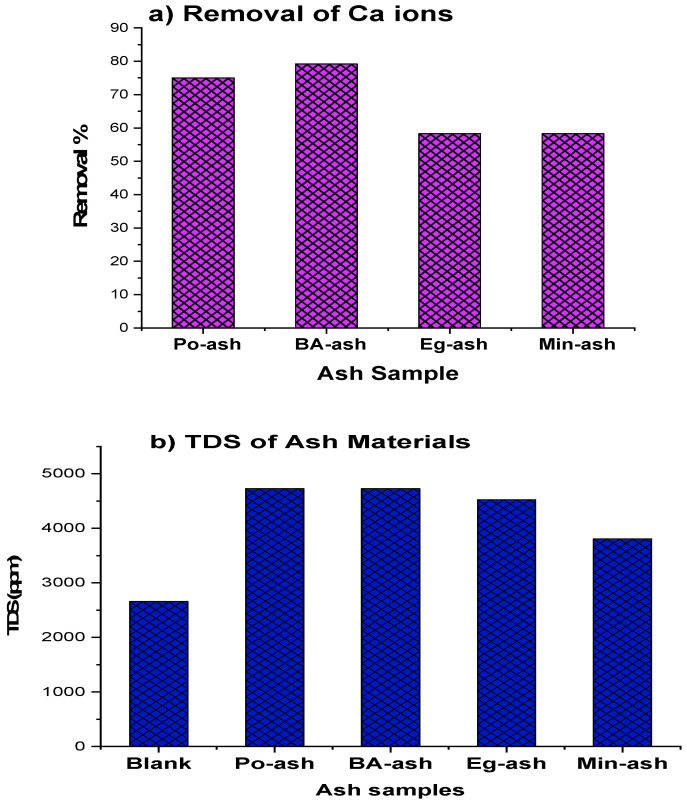
Comparison of the examined ash samples: (**a**) %Ca removal; (**b**) TDS in solution.

Based on data in Table 4, the tested ashes displayed a great affinity for diminution of Ca ions from the solution. The elimination efficiency was above 75% for Po-ash and Ba-ash, while Eg-ash and Min-ash were approximately 58% at extremely high initial Ca content (1000 ppm) as illustrated in Figure 5a. Only 10 g/L of ash samples were sufficient to eliminate 58–75% of the total significantly hard water solutions. In addition, all examined ash materials increased the pH values for treated feed solutions without increasing the number of hydroxyl ions. Po-ash, BA-ash, and Min-ash raised the pH up to 9.6, while Eg-ash increased the pH to 8.8, which indicated that the basic nature of the ash materials was in agreement with other researchers [25,26,49,50].

Regarding conductivity and TDS measurements from Figure 5b and Table 4, the results showed a 43% increment after adding a 10 g/L dose of ash materials for Po-ash, BA-ash, and Eg-ash, while Min-ash showed only a 30% increment. This may be the result of existing soluble inorganic salts, e.g., KCl, KHCO_3_, NaCl, and NaHCO_3_, which were confirmed by XRD analysis. Other published studies displayed increments in TDS or conductivity in the same way [26,39]. Bearing in mind that Na^+^ and K^+^ ions remaining in the feed solution cannot form scale, they are also much easier to be removed with the employment of an RO system in order to meet the standards allowed for drinking water [51].

#### Changes in Water Quality by Increasing the Amount of Agro-Ash in Pure Distilled Water

First, we needed to study the nature and chemical properties of treated water after adding a definite amount of the tested ash materials, measuring the released number of cations and anions in aqueous solutions to explain the proper mechanism for removing Ca ions from solutions. The preliminary dissolution of the tested ash materials in distilled water was evaluated by mixing 1 g of agro-ash in 100 mL of distilled water with a contact time of 1 h. The results presented in Table 5 show the water quality after treatment with agro-ash. The reported numbers show an increase in the level of pH values up to nine, indicating the basic character of the ash samples. All examined agro-ash reflected high conductivity (approximately 5.3–8.7 mS/cm) and increments in TDS measurements (more than 2.6 g/L) for all tested samples. The large majority of cations (i.e., K^+^, Na^+^, Ca^2+^, and Mg^2+^) was indicated, mainly high amount of K^+^ as well anions, particularly Cl^−^, SO_4_^2−^, CO_3_^2−^, and HCO_3_^–^. However, the amounts of sodium were small compared to the potassium content.

The remarkable aspects of the tested ash materials were their significant amounts of carbonate and bicarbonate, which make them valuable resources. Po-ash and Eg-ash released the highest amounts of HCO_3_^−^ at 410 and 310 ppm for the presence of a mixture of KHCO_3_ and NaHCO_3_. While Min-ash, BA-ash, and Eg-ash released a high quantity of CO_3_^2−^ in agreement with the previous mineralogical analyses of the XRD patterns (see Section 3.1.): Na_3_Mg(PO_4_)CO_3_, Ca_7_(Si_6_O_18_)(CO_3_)(H_2_O)_2_, Ca_5_(SiO_4_)_2_CO_3_, and CaCO_3_.

### 3.5. The Proper Mechanism for the Removal of Ca Ions from an Aqueous System

Details derived from all analysis techniques of the burned ashes about their definite compositions helped in the prediction of the appropriate mechanism for removal of Ca ions from the aquatic system. Data derived from the XRD patterns indicated various soluble and non-soluble minerals such as carbonate and bicarbonate structures: nahcolite, kalicinite, bradleyite, calcite, scawtite, and spurrite. All alkali metals’ carbonates or bicarbonates are highly soluble in water at a lower pH values, while other types of carbonates, such as CaCO_3_(s), are hardly soluble at higher pH levels [50]. Therefore, the existence of enormously soluble alkali bicarbonates (NaHCO_3_ and KHCO_3_) was responsible for the rapid augment in pH values and the formation of metal hydroxide at high rates according to the reactions described below [8,17,52]. The successive Equations (2)–(4) are:

MHCO_3_ (s) + 2H_2_O (l) → 2MOH (aq) + H_2_CO_3_ (aq) (2)

where M = Na^+^ or K^+^.

HCO_3_^−^ + OH^−^ → CO_3_^2−^ + H_2_O (3)

Ca^2+^ (aq.) + CO_3_^2−^ (aq.) → CaCO_3_ (s) ↓ (4)

The predicted mechanism for the removal of calcium ions can be explained by two factors. The first dominant one came from hydrolysis of various soluble carbonates and bicarbonates present in the ash materials which can (i) enhance hydroxyl ion formation leading to dramatic increases in the pH of the solution, as demonstrated in Table 4 and Table 5, which can form sodium and potassium hydroxides that play the same role as lime in water softening as a source of alkali. (ii) The existence of carbonates and bicarbonates in the solution were easily converted to noncarbonated Ca ions in the solution to an insoluble form of CaCO_3_ [52]. (iii) After increasing the pH of the solution, the bicarbonate radicals easily react with hydroxyl ions to form a carbonate radical that can react with Ca^2+^ [17]. The second mechanism may enhance the reduction in Ca^2+^ ions due to the adsorption role of insoluble inorganic minerals existing in the composition of agro-ash such as charcoal, silicates, and CaCO_3_ [50]. Precipitation by alkali/carbonate sources is a familiar method used for reducing the hardness of water [11,14,52,53,54]. Mg(OH)_2_ was used as an alkali source derived from the brine pre-treated waste of soda ash plant mixed with the flux of CO_2_ for water decalcification by precipitation of CaCO_3_ [17]. In addition, ash derived from coal plants is used as strong alkali material, showing a high pH value in the range 10–13, and it was utilized as a chemical precipitant in an aqueous solution [55]. Moreover, ashes derived from bamboo, rice husk, banana rind, and banana pseudostems were used as a precipitant for iron removal from groundwater [25]. Furthermore, NaHCO_3_ and limestone were studied in a previous work to increase the alkalinity in aqueous solutions and facilitate precipitation of Cd, Pb, Zn, Ni, Cu, and Cr ions from solutions [56]. The precipitation process is easily available in high basic solutions for the removal of Ca and Sr ions by lime and bicarbonate [57] and for Cu ions by using ash from coal-fired power plants [56].

#### Analysis of the Residue Agro-Ash during the Precipitation Process

**Figure 6:**
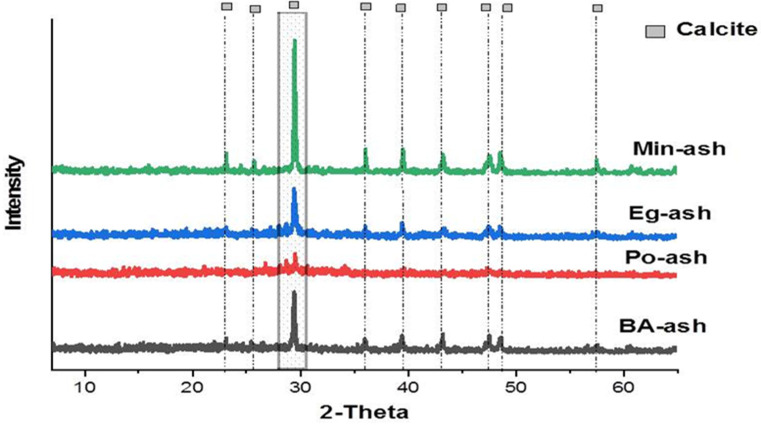
XRD of CaCO_3_ precipitating in the residue of agro-ash material presenting in the calcite phase.

The above mechanism (Section 3.5.) was confirmed by analysis of the residual ash materials after mixing the agro-ash with a feed solution containing Ca^2+^. The formation of CaCO_3_ was indicated by XRD analysis that pointed to the presence of synthetic CaCO_3_ in the form of a calcite structure as illustrated in Figure 6. The XRD spectrographs of synthetic calcite (CaCO_3_) demonstrated eight distinctive peaks according to JCPDS card no. 05-0586. The illustrating crystal planes were (012), (104), (110), (113), (202), (018), (116), and (122), and they appeared at 2θ = 22.99, 29.36, 35.94, 39.52, 43.32, 47.55, 48.60, and 57.54°, while their diffraction lines were observed at d = 3.84, 3.02, 2.48, 2.27, 2.08, 1.91, 1.87, and 1.60 [58]. According to the results obtained from the XRD peaks, only one phase structure of CaCO_3_ was formed as calcite, but the other phases, such as vaterite or aragonite, cannot form under these conditions [14,58].

### 3.6. Influence of the Initial Calcium Concentrations on the Removal Process

**Figure 7:**
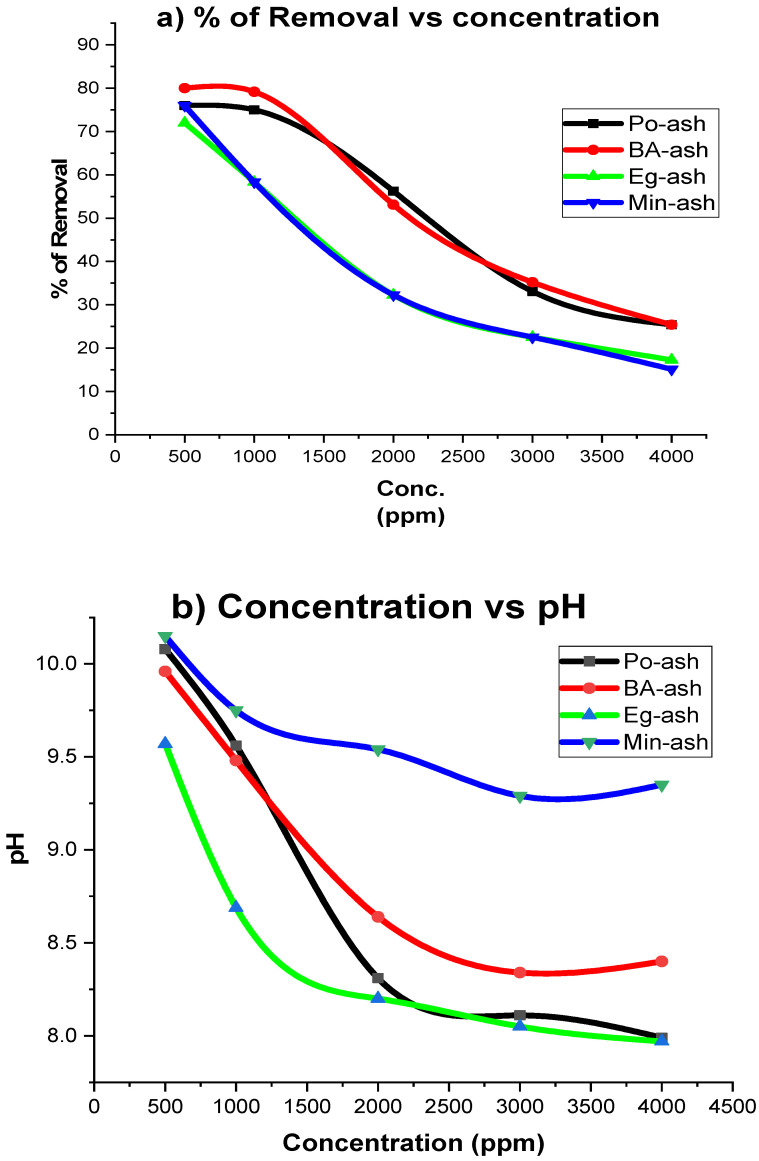
Influence of the initial concentrations against the removal of Ca ions in (**a**) versus the pH in (**b**) of the solution.

Reduction of Ca (II) as a hardness ion is significantly controlled by the initial concentrations of aqueous resources. The influence of initial calcium ion concentration on the removal process was examined at a fixed ash mass (10 g/L) and is revealed in Figure 7a. The increasing concentration of Ca ions from 500 to 4000 mg/L showed a noticeable decrease in the elimination affinity. The removal of Ca ions reached nearly 80% (at an initial concentration of 500 ppm) for all the tested ash materials. However, increasing the initial concentration above 3000 ppm showed a dramatic decrease in removal efficiency to 20%. The reason for such behavior may be explained by the limited activity of ash materials at a fixed dose (10 g/L), and the used dosage was not adequate for the reduction of high-level contents of Ca ions [18,44]. Hence, with an increase in the initial Ca^2+^ ions, more ash must be added to release more hydroxide and carbonate ions to enhance the precipitation of Ca^2+^ ions from the solution [25]. A noteworthy, good relationship between the initial concentration of Ca ions and pH of the feed solution was observed. For a fixed dose of 10 g/L of tested ash samples, increasing the initial Ca ion content (from 500 to 4000 ppm) resulted in a decline in the pH of the solution from 10 to 8.0 as demonstrated in Figure 7b. This decrease in the final pH value can be explained by the dissolution of the high level of CaCl_2_.6H_2_O in a solution that lowered the pH of the aqueous solutions and became more acidic, less than seven [59]. Therefore, the added amount of agro-ash consumed part of them to neutralize these acidic solutions.

### 3.7. Influence of the pH of the Solution

**Figure 8:**
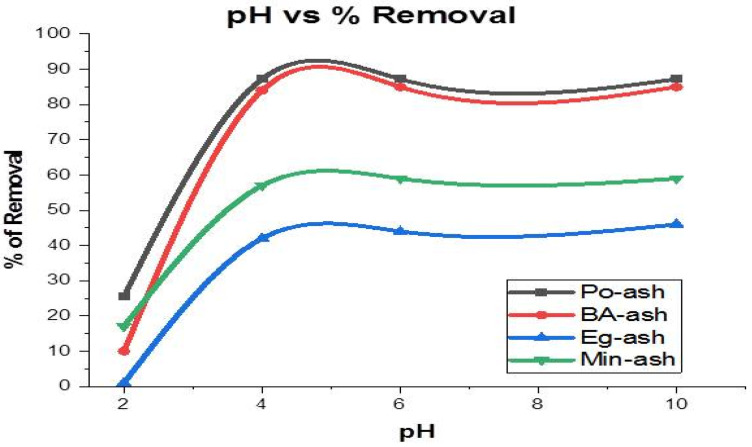
The influence of pH on the Removal of Ca ions.

Removal of metal ions are strongly governed by pH values in water and wastewater engineering. The influence of changing pH values on the removal of Ca ions with the tested ash materials was investigated and is illustrated in Figure 8. All examined ashes showed augments in final pH values of the feed solution from faintly alkaline (pH 8.2) to an extremely high basic medium (pH 9.8). The tested ash substance increased the pH values of treated water in the following order: Min-ash > BA-ash ≈ Po-ash > Eg-ash. Similar observations were reported for various fly ash and biochar materials exhibiting a basic nature and an increase in the alkalinity of the aqueous solution [16,25,26,44,50]. With the increasing pH of the feed solution, the reduction rate for Ca ions was enhanced. In pH values lower than three, the reduction did not exceed 25% of the total Ca content. Decreasing the pH values of the aqueous solution to less than 4.3 converted bicarbonate into carbonic acid, while the increasing the alkalinity of the solutions helped in the conversion of bicarbonates into carbonates and, consequently, the easy removal of calcium as CaCO_3_ [54]. Moreover, the precipitate of calcium carbonate began to dissolve in acidic conditions [17]. Thus, a fast removal of Ca ions was obtained beyond pH 5 and the increasing onset of the precipitation process [50]. The appropriate pH for the precipitation of calcium carbonate was 9.5 [14], and the carbonation process for the precipitation of CaCO_3_ was not stopped as soon as the pH values did not drop to a pH lower than seven. Hence, a large amount of ash material is recommended to neutralize the acidic nature of feed solutions. The increasing pH value from 9.5 to 10.5 may also help in removing other heavy metals, such as iron, manganese, lead, copper, zinc, and arsenic, by precipitation [60].

### 3.8. Studying the Mass of Ash Materials

**Figure 9:**
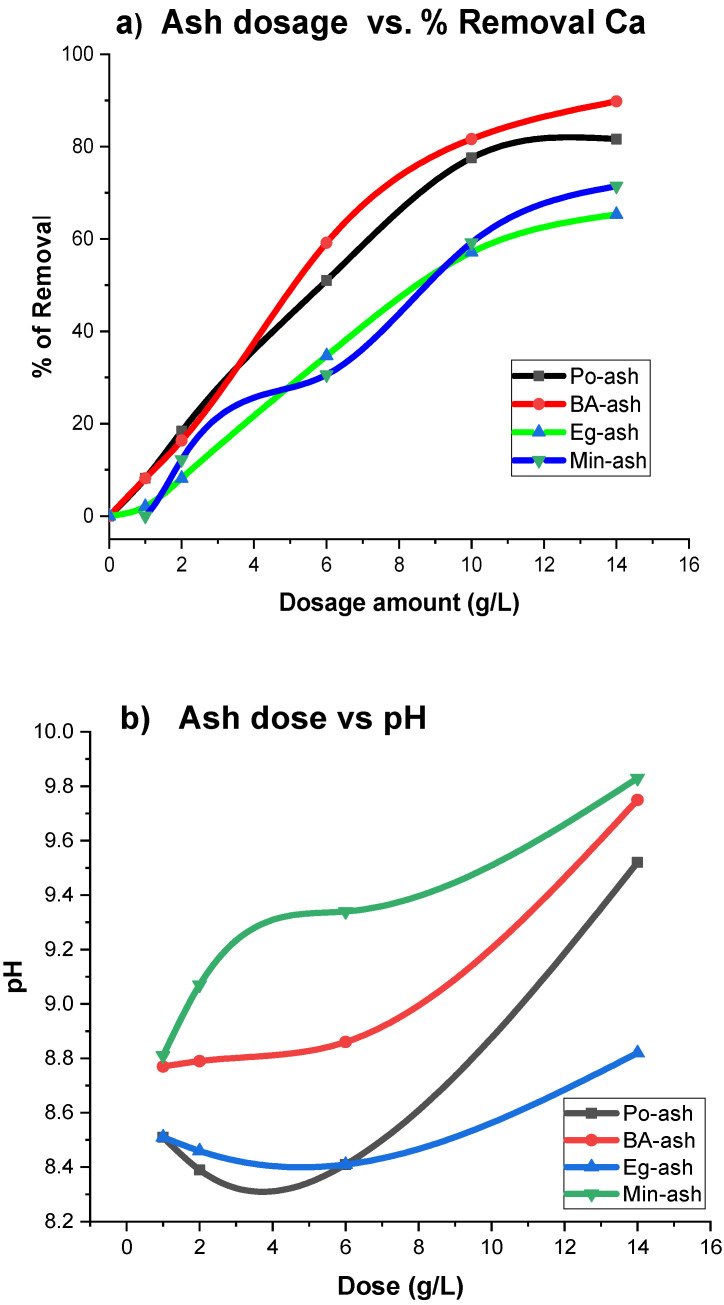
(**a**) Influence of ash dose versus Ca removal; (**b**) influence of ash dose versus pH.

Adsorbent dosage is an important parameter for determining the capacity of the materials; therefore, we can ascertain the optimum dose for the maximum extent of removal. Figure 9a illustrates the ability of examined agro-ash dosages on the removal ability of calcium ions. It can easily be pointed out from the obtained results that the percentage of Ca ion removal increased with an increase in the used amount of ash materials. The increased dose, from 2 to 14 g/L, increased the efficiency of Ca removal from 8% to approximately 82% in for Po-ash and BA-ash and 66% for Min-ash and Eg-ash. A sharp increase in the removal efficiency after a dose of 6 g/L was observed. Therefore, the employed dosage of ash at 10 g/L was quiet enough to achieve 80% removal efficiency for Po-ash and BA-ash, while Min-ash and Eg-ash provided only 58% Ca removal. Higher amounts of ash provide more soluble carbonate and bicarbonate that raises the pH of the solution and enhances the precipitation conditions of Ca ions [25,54]. Moreover, a remarkable enhancement in the pH of the aqueous solution was accompanied by an increasing amount of added ash mass as presented in Figure 9b. This large increase in pH values of the solution was due to the rapid dissolution of all soluble alkali bicarbonate and carbonate presented in the tested ash compositions [25,50]. This increase in pH during the softening experiment was similar to the increase in the action of lime [14], even though by adding a small quantity of ash, the pH values increased to 8.5. Furthermore, an assessment of the increase in the total TDS and conductivity measurements was indicated (as presented in Table 3) due to the basic character of the tested ash materials. Utilization of the (RO) system was needed for further purification of the water from soluble Na^+^ and K^+^ cations to meet the quality standards of drinking water [32].

### 3.9. Contact Time

**Figure 10:**
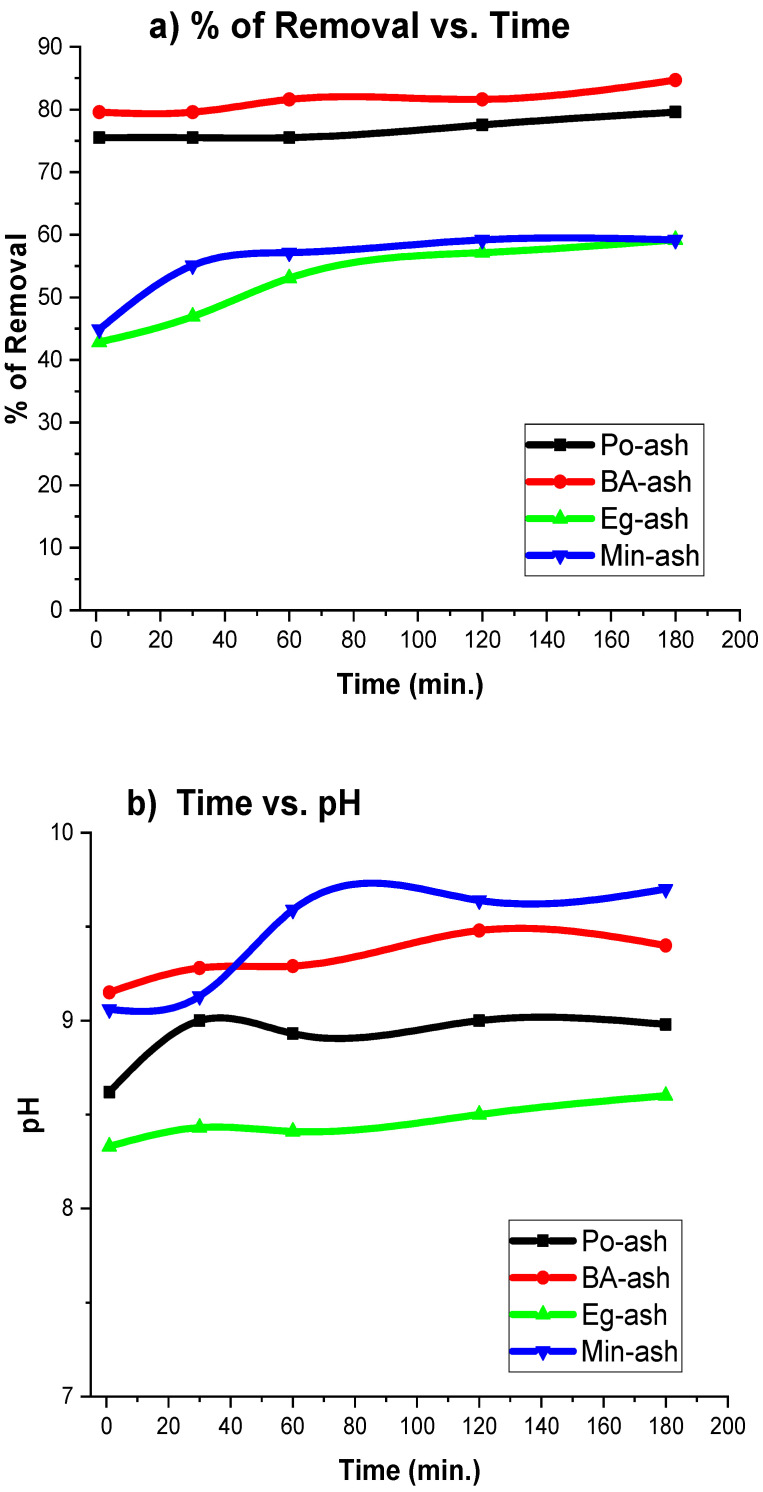
(**a**) Contact time versus removal of Ca ions; (**b**) contact time versus pH of the solution.

The elimination rate of Ca ions from solutions is considered an essential factor for designing reactors for effective practical purposes [61]. The results of studying the effect of contact time between the ago-ash and feed solution are shown in Figure 10a. Another extra feature of using agro-ash materials (besides their application as an alternative method to the lime/soda ash method) is their rapid elimination of major hardness ions (Ca^2+^) within 5 min, especially when compared to other methods. No more than ten minutes were needed to complete the removal of Ca ions as shown in Figure 10a, even though other published studies have demonstrated that more time was required for attending to the equilibrium of Ca ion removal. The equilibrium times were 240 min by modified pumice [39], 180 min by white clay [17], 24 h by ureolytic microbiological carbonate precipitation [6], 10 min by [4,4′-isopropylidenebis (phenoxyacetate)] [11], 3 days by utilization of the microbial carbonate precipitation process [9], 10 min by using wheat straw and rice husk ashes [16], and 90 min by modified bentonite [33]. The fact that there was no need to increase the contact time of the examined agro-ash with solutions by more than 30 min in most cases of Po-ash, BA-ash, Eg-ash, and Min-ash was considered. In addition, no significant increment in the pH of solutions with an increase in the contact time was observed as illustrated in Figure 10b. The feed solutions entered a steady state in approximately 60 min.

### 3.10. Comparison between Common Precipitation Methods and Tested Agro-Ash

Several laboratory examinations were carried out to compare various conventional methods with the examined agro-ash for removal of Ca ions, bearing in mind that Ca ions are the primary cation that creates scales when presented in water samples. Hydrated lime, soda ash, and caustic soda are considered the most cost-effective substances frequently used in water plants, and the required lime dosages for water softening are approximately 100–200 mg/1 [60]. In this study, five commonly substances were utilized: NaOH, Ca(OH)_2_, NaHCO_3_, Na_2_CO_3_, and CaO and the two mixtures of CaO and Na_2_CO_3_ and Ca(OH)_2_ and Na_2_CO_3_ compared with the tested ago-ashes. The results are demonstrated in Table 6, and the efficiency of Po-ash and BA-ash showed higher calcium removal by approximately 75% more than NaHCO_3_ or Na_2_CO_3_ (60%) in high initial concentration of Ca ions of 1000 ppm. Moreover, the Eg-ash and Min-ash also showed good removal of Ca at approximately 58%, which was very close to the results of the treatment with NaHCO_3_ or Na_2_CO_3_. The tested agro-ash materials used in the water treatment were accessible and free of charge, and they were cost-effective and environmentally benign materials unlike other chemical precipitants, such as NaOH, Ca(OH)_2_, NaHCO_3_, Na_2_CO_3,_ and CaO, which must be purchased. There was also no necessity to further use chemicals in the treatment process or make any modifications to the agro-ash materials. This made the demand for agro-ash more significant than conventionally used lime, limestone, soda ash, or any other industrial chemicals which are expensive [50]. Other researchers studied the reuse of different alkali sources, such as Mg(OH)_2_ and CaCO_3_, for the pre-treatment of seawater [17] using basic oxygen furnace slag (BOF) and the soda ash–lime method to treat acid mine drainage [51]. In addition, Namibian charcoal ash from acid mine drainage was used in the elimination of metals and sulphate ions [50], while boiler fly ash (RBFA) was used to remove Pb, Cd, and Zn ions from the cycle of kraft pulp mills [62]. Other agricultural ash materials, such as wheat straw and rice husk ash, were used to eliminate hardness ions from the solution [16].

### 3.11. SWOT Analysis for Using the Tested Agro-Ash as an Alternative Method

The SWOT analysis was created by Albert S. Humphrey as a simplistic tool for evaluating various technical choices and assessment methods. This approach examines both a future technology’s internal strengths and weaknesses and external environmental opportunities and threats in order to determine its flaws [63]. SWOT analysis assesses the positive and negative perspectives of different variables on the system’s long-term sustainability. The SWOT analysis (Table 7) was used in this study to pay attention to the worth of producing ash material from agro-waste using combustion and gasification processes. It was revealed that the tested biomass had the least expensive impact on water decalcification among the other biomasses.

## 4. Conclusions

Scale formation is one of the challenges that face the industrial sector, and it is strongly related to economic activities and water services. Examined agro-ash in this work showed a great ability to reduce Ca ions from aquatic resources. The elimination efficiency was above 75% for Po-ash and Ba-ash, while Eg-ash and Min-ash showed approximately 58% at extremely high initial Ca contents (1000 ppm). The extraordinary features of the tested agro-ash materials were significant levels of carbonates and bicarbonates, which make them valuable resources. Thus, agro-ash may be a green alternative method to employing lime/soda ash or caustic soda. The ash materials are not toxic substances; they are by-products of natural plants. Only 10 g/L of ash samples was sufficient to remove 58–75% of the total very hard water solutions. The efficiency of Po-ash and BA-ash for the decalcification process exhibited higher removal ability (75%) compared to consumption of NaHCO_3_ or Na_2_CO_3_ (60%). Moreover, there was no necessity for modifications to the agro-ash materials or the utilization of additional chemicals. Collecting and reusing agro-ash is a good method for obtaining a clean environment free of pollution. Overall, elimination of fouling and scaling is a vital issue in remediation of industrial wastewater before using an RO system to prolong the membrane’s lifespan. Sustainable use of waste and expansion in green eco-friendly substances may help to save our resources and deflate treatment costs.

## Figures and Tables

**Table 1:** XRD analysis of the constitutions of the tested agro-ashes, (Po-ash is Potato peel Ash, BA-ash is Banana peel Ash, Eg-Ash is Eggplant ash, and Min-Ash is Mint stem ash).

Minerals Phase	JCPDS Card	Chemical Structure	Compositions of Agro-Ash Samples %			
			Po-ash	BA-ash	Eg-ash	Min-ash
Sylvite, syn	73-0380 (C)	KCl	6.0	15.5	11.5	7.4
Kalicinite	70-1167 ((D)	KHCO_3_	11.7	-	19.8	-
Langbeinite	72-1206 (C)	K_2_Mg_2_(SO4)_3_	16.0	-	27.4	33.2
Nahcolite	21-1119 (D)	NaHCO_3_	22.3	-	24.2	45.2
Bradleyite	22-0478 (I)	Na_3_Mg(PO_4_)CO_3_	1.0	-	17.1	-
Halite, syn	05-0628 (*)	NaCl	5.1	9.8	-	-
Scawtite	81-1918 (C)	Ca_7_(Si_6_O_18_)(CO_3_)(H_2_O)_2_	37.9	43.5	-	-
Spurrite	13-0496 (I)	Ca_5_(SiO_4_)2CO_3_	-	31.3	-	-
Calcite	72-1652 (C)	CaCO_3_	-	-	-	14.1

**Table 2:** Elemental analysis (EDX) of the tested ash materials.

Elements Composition	Weight %			
	Po-ash	BA-ash	Eg-ash	Min-ash
C	22.44	11.26	25.72	14.49
Na	0.27	nd *	0.2	0.96
Mg	0.36	1.38	0.19	1.28
Al	0.18	nd *	nd *	0.33
Si	0.34	3.87	nd *	0.71
P	0.44	0.91	0.19	1.38
S	0.24	0.53	0.05	0.69
Cl	1.3	5.7	1.32	6.05
K	10.23	29.83	2.73	20.22
Ca	0.27	2.25	nd *	4.03
Fe	nd	nd *	nd *	0.35
O	63.9	44.28	69.6	49.52
Total	100	100	100	100

* Not detected.

**Table 3:** FTIR spectra assignment for raw and ash materials.

Functional Groups	Wavenumber cm^−1^		Reference
	Raw Materials	Ash Materials	
OH Stretching	3401	-	[32,35]
C–H bond of CH_2_ group	2915	-	[32,36,37,38]
O–H bending	1639	1652	[39,40,43]
Carboxyl (–COOH) groups	1749	-	[32,36,37]
CO stretching bond of CO_3_^2−^	1400	1450	[26,38,44,45]
Si–O–Si asymmetric Or SiO_4_ ^2−^ group	1064	1061	[26,32,35,46,47]
P–O stretching PO_4_ ^2−^ group	-	1104	[38,46]
M–O bond	-	863	[42,48]
Bending S–O of SO_4_ ^−2^	-	617	[38,46]

**Table 4:** Changes in water quality after adding 10 g/L of ash materials in 1000 ppm Ca ions.

Samples	Final pH	Conductivity (mS/cm)	TDS (ppm)	% Removal
Po-ash	9.5	9.2	4729	75
BA-ash	9.6	9.2	4729	79.2
Eg-ash	8.7	8.8	4524	58.3
Min-ash	9.7	7.4	3806	58.3
Blank Ca^2+^: 1000 ppm	7.7	5.19	2659	-

**Table 5:** Quality changes in 100 mL of distilled water by adding 1 g of the tested ash.

Parameters	Units	Dist. Water	Po-ash	BA-ash	Eg-ash	Min-ash
Total hardness	ppm	-	100	80	80	140
pH	-	6.9	9.7	10.3	10.6	10.7
Conductivity	mS/cm	0.008	6.6	6.8	8.7	5.3
Na^+^	ppm	-	54	6	99	196
K^+^	g/L	-	1.64	1.64	2.34	1.35
Fe^2+^	ppm	-	nd	nd	nd	nd
Mn^2+^	ppm	-	0.1	0.1	0.1	0.1
Cl^−^	g/L	-	0.74	0.45	0.26	0.22
SO_4_^2−^	g/L	-	0.44	0.09	0.37	0.26
CO_3_^2−^	g/L	-	1.62	3.0	2.88	3.66
HCO_3_^−^	g/L	-	4.09	1.40	3.11	0.43

**Table 6:** Comparison of common chemical precipitation methods with agro-ash.

Reagents	pH	Cond. (mS/cm)	TDS (g/L)	% Removal
0.2% NaOH	12.3	13.4	6.73	10%
0.2% NaHCO_3_	7.87	5.99	2.99	60%
0.2% Na_2_CO_3_	8.22	5.51	2.75	72%
0.2% Ca(OH)_2_	12.2	12.7	6.18	Increment
0.2% Ca(OH)_2_ + 0.2% Na_2_CO_3_	11.6	7.04	3.52	60%
0.2% CaO + 0.2% Na_2_CO_3_	12.3	11.2	5.62	10%
PO-ash (0.5 g/L)	9.5	9.2	4.72	75
BA-ash (0.5 g/L)	9.6	9.2	4.72	79%
Eg-ash (0.5 g/L)	8.7	8.8	4.52	58%
Min-ash (0.5 g/L)	9.7	7.4	3.80	58%
Blank (1000 ppm CaCl_2_)	7.7	5.19	2.65	-

**Table 7:** SWOT analysis for using green agro-ashes.

SWOT Analysis	
**Strength**	**Weakness**
Tested agro-ash materials have significant levels of carbonates and bicarbonates, which make them alternative resources. In the elimination of hardness in water purification, agro-ashes are an effective substitute for soda ash and CaO materials. The use of agro ashes is a simple way to eliminate scale formation in the industrial sector. The ability of ash compounds was successful even when the initial Ca level was extremely high (1000 ppm). The ash products include no hazardous chemicals. Collecting and reusing agro-ash will provide a safe method for achieving a pollution-free environment. Low operating and maintenance costs. Agro-ash eliminated calcium ions from water in a very short time.	TDS levels were 30 percent higher in treated water than in untreated water. Scales will not form in the presence of leftover Na and K ions in the solution. An RO unit is needed to remove Na and K ions.
**Opportunities**	**Threats**
Promotion of ash derived from agro-wastes as alternative substances to precipitate heavy metals from water resources. Substitution of Ca(OH)_2_ by natural material. Amendments prevent rubbish from accumulating and landfills from expanding. Developing in-depth studies in the field of waste management. Applying the 3R strategy (reduce, reuse, and recycle). Any other green biomass appears to be acceptable for agro-ash production.	Concerns about air pollution produced by the combustion of agricultural waste.

## Data Availability

Did not report any data.
